# Improved accuracy in flow mapping of congenital heart disease using stationary phantom technique

**DOI:** 10.1186/1532-429X-11-52

**Published:** 2009-12-10

**Authors:** Thomas A Miller, Andrew B Landes, Adrian M Moran

**Affiliations:** 1 Department of Pediatrics, Maine Medical Center, 22 Bramhall St, Portland, ME, USA; 2 Department of Radiology, Maine Medical Center, 22 Bramhall St, Portland, ME, USA; 3 Department of Cardiology, Maine Medical Center, 22 Bramhall St, Portland, ME, USA

## Abstract

**Background:**

Flow mapping by cardiovascular magnetic resonance has become the gold standard for non-invasively defining cardiac output (CO), shunt flow and regurgitation. Previous reports have highlighted the presence of inherent errors in flow mapping that are improved with the use of a stationary phantom control. To our knowledge, these studies have only been performed in healthy volunteers.

**Results:**

We analyzed the variation in flow measurements made with and without stationary phantom correction in 31 patients with congenital heart disease. Variation in stroke volume (SV) measurements was seen in all vessels across all patient groups. The variation was largest when analyzing the right ventricular outflow tract (RVOT), with a range of absolute differences in SV from 0.2 to 70 ml and in CO from 0.02 to 4.8 L/min. In patients with repaired Tetrology of Fallot (ToF), the average ratio of pulmonary to systemic blood flow (Qp:Qs) was 1.18 without and 1.02 with phantom correction. Without performing phantom correction, 23% of the repaired ToF patients were classified as having a residual shunt as compared to 0% when flow mapping was performed with phantom correction. Similarly, in patients with known atrial level shunting (ASD/PAPVR) 20% of patients had no shunt when flow mapping was performed without phantom correction as compared to 0% with phantom correction. In patients with bicuspid aortic valves (BAV), the differences in the regurgitant fraction between measuring flow with and without phantom correction ranged from 0 to 30%, while the regurgitant fraction in the RVOT of ToF patients varied by as much as 31%.

**Conclusion:**

The impact of inherent errors in CMR flow mapping should not be underestimated. While the variation across a population may not display a significant trend, for any individual patient it can be quite large. Failure to correct for such variation can lead to clinically significant misinterpretation of flow data. The use of the stationary phantom correction technique appears to improve accuracy both in normal patients as well as those with congenital heart disease.

## Background

Quantifying blood flow is necessary in guiding management for patients with congenital heart disease. Flow mapping by cardiovascular magnetic resonance (CMR) provides a non-invasive modality to measure cardiac output, shunt flow and regurgitation [1]. Phase contrast CMR correlates with invasive measurements of left-to-right shunts [2] and has been shown as an effective alternative to routine catheterization for evaluation of selected patients prior to bidirectional Glenn operation [3]. Furthermore, CMR interstudy reproducibility is reportedly higher than that of echocardiography [4]. Despite its advantages, flow mapping by CMR has both technical and clinical sources of error including sampling rate, partial volume averaging, sub-optimal velocity encoding selection, flow-turbulence, aliasing and intra-study heart rate changes [5]. These errors are well recognized and attempts are made to minimize their impact during data acquisition and post-processing analysis. When these sources of error are unable to be controlled, comments are made in final reports regarding their effects on study interpretation.

Flow mapping errors also occur secondary to local eddy currents as well as concomitant field gradients which create a baseline velocity or background *flow *which may go unrecognized [6-8]. While CMR scanners automatically correct for concomitant gradient effects during phase contrast image reconstruction, the presence of eddy-currentinduced fields can still cause substantial errors. The magnitude of such errors depends on several imaging parameters, including where the vessel is relative to the isocenter, the imaging planes, and the velocity encoding gradient strength [7].

Seemingly small velocity errors are greatly magnified when integrated across the cross section of a vessel to calculate blood flow. Several approaches are used to compensate for background *flow*. These include the analysis of a background region of interest in stationary tissue adjacent to the vessel of interest, as well as using more distant stationary tissue and estimating phase offsets using linear or higher order interpolation [9-11]. Errors can also be controlled by using a stationary phantom technique. This technique involves repeating the same imaging sequence on a stationary fluid phantom.

Background *flow *can be measured in the exact same location in the scanner as the vessel of interest. This method has been previously shown to improve the accuracy of flow measurements as demonstrated by improved pulmonary to systemic blood flow ratios in healthy volunteers [7].

To our knowledge, the effect of stationary phantom correction on flow mapping in congenital heart disease has not been reported. In this study we investigate whether correction of background *flow *with the use of stationary phantom techniques can have a significant effect on flow mapping and subsequent analysis of cardiac physiology in patients with Tetralogy of Fallot (ToF), bicuspid aortic valves (BAV) and known atrial level shunting from either atrial septal defects or partial anomalous pulmonary venous return (ASD/PAPVR).

## Methods

### Patient Selection

We reviewed all patients that had phase velocity flow mapping by CMR for the diagnosis of repaired ToF, BAV or atrial level shunting from ASD or PAPVR between January 2007 and August 2008. All flow mapping was retrospectively reviewed with and without phantom correction, blinded to initial technical and clinical reports. After blinded analysis was complete, the technical and clinical reports were reviewed and patients were excluded from further analysis based on CMR study limitations. All imaging was performed when clinically indicated.

### MRI Acquisition and Analysis

All patients were imaged using GE 1.5T Signa HDX systems using GE 15.0 M4 software. Phase contrast images were acquired perpendicular to the long axis of the proximal ascending aorta, proximal main pulmonary artery, proximal right and proximal left pulmonary arteries. Every effort was made to ensure the vessel of interest was as close to isocenter as possible and a minimum velocity encoding gradient strength (Venc) to avoid aliasing was chosen. Breath held images were acquired using the commercial FastCine phase contrast pulse sequence which uses continuous, uninterrupted radiofrequency excitations, prospectively gated phase encoding, and retrospectively gated image reconstruction. The 'flow analysis' setting was applied during image acquisition so that the pulse sequence automatically compensated for the concomitant gradient effects. Acquisition parameters included, TR 7.6-8.0 ms, TE 3.1-3.5 ms, matrix 256 × 128, FOV 480 mm × 384-480 mm, bandwidth 31.25 kHz, slice thickness 8 mm. Number of segmented k-space lines was adjusted for heart rate (HR) per manufacturer recommendations as follows: HR<60 = 8, HR 61-94 = 6, HR>95 = 4. After the flows were acquired, background *flow *was measured by imaging a phantom with identical phase contrast imaging parameters. A period of 5-10 minutes occurred from the time of phantom placement to image acquisition to avoid fluid movement within the phantom. All postprocessing was performed by two individuals (AMM and ABL) using commercially available software (ReportCard 3.0 GE Medical Systems, Milwaukee, WI). Phase velocity flow analysis was performed using standard region of interest (ROI) method. To correct for background *flow *a ROI of the same size and location was automatically placed on the corresponding phantom image [7]. Corrected flow was calculated by subtraction on an image by image basis.

### Data and Statistical Analysis

Data and statistical analysis was performed with Microsoft Excel (Microsoft, Redmond, WA) and Prism (GraphPad, San Diego, CA) software. Cardiac output (CO) was calculated for each vessel as SV multiplied by the heart rate (HR) at the time of flow mapping. Differences in SV and CO were always calculated as the measurement without phantom control minus the measurement with phantom control. The ratio of pulmonary to systemic arterial flow (Qp:Qs) was calculated with both SV and CO using flow through the BPAs to define pulmonary blood flow. Regurgitant fraction was calculated as retrograde SV divided by antegrade SV.

## Results

### Patient Selection and Internal Control

During the time of our study there were 20, 11 and 10 patients evaluated by CMR for ToF, BAV and atrial level shunting, respectively. In the ToF group, 4 patients were excluded from all analysis due to arrhythmias making gating inaccurate, 2 patients were excluded from analysis involving the branch pulmonary arteries (BPAs) secondary to heart rate variability and BPAs moving in and out of plane, 1 patient was excluded from analysis involving aortic flow secondary to artifact from sternal wires. No patients were excluded in the BAV group. In the ASD/PAPVR group, one patient was excluded from analysis involving the right ventricular outflow tract (RVOT) secondary to incorrect plane selection. Age at time of study was 1-77 years (median, 23). Inter- and intraobserver correlation for stroke volume (SV) measurements for the two individuals who performed all of the post processing analysis was 0.98 and 0.99 while the mean difference was 1.9 ± 5 ml and -1.9 ± 4 ml, respectively.

### Stroke Volume

**Figure 1:**
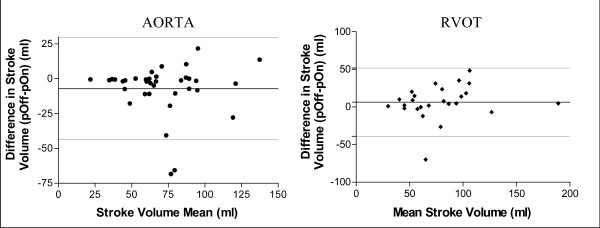
**Bland-Altman Analysis of Stroke Volume Measurements with and without Phantom Correction**. Scatter plots show the mean stroke volume compared to the difference in stroke volume for measurements with and without phantom correction. Each plot represents a single patient. The plots include patients across all three diagnostic groups. The solid horizontal line represents the mean of the differences in SV, while the dotted lines represent 2 standard deviations of that mean in each direction. RVOT = Right ventricular outflow tract. pOff-pOn = SV measured without phantom correction minus SV measured with phantom correction.

We found that the correlation between flow measurements made with and without phantom correction was highly variable. The correlation coefficients for SV measurements in each vessel are shown in table 1. In each diagnostic group, there was a weaker correlation for flow measurements obtained in the aorta and RVOT than the BPAs. This trend was also present when combining flow data across diagnostic groups to yield the following linear regression curves for each vessel. For the aorta, *y *= 0.75*x *+ 11.5, r^2 ^= 0.59; for the RVOT, *y *= 0.90*x *+ 14.2, r^2 ^= 0.64; for the right pulmonary artery, *y *= 0.97*x *+ 0.18, r^2 ^= 0.98; for the left pulmonary artery, *y *= 1.01*x *- 2.4, r^2 ^= 0.81. Table 2 highlights how markedly different the flow measurements were between the two methods for any given patient. SV changes of 68 and 70 ml as reported in table 2 correlate to differences in CO of 3.4 and 4.8 L/min, respectively. Similar to our findings with correlation, absolute differences in SV measurements between the two methods were more drastic in the aorta and RVOT than they were in the BPAs (table 2). A Bland-Altman analysis [12] of differences in SV between the two methods, shows no trend in variation based on the magnitude of the SV (figure 1).

**Table 1:** Correlation coefficient of stroke volume with and without phantom correction.

		Vessel SV Correlation			
		**Aorta**	**RVOT/MPA**	**RPA**	**LPA**

Patient Group	All	0.77	0.80	0.99	0.90
	
	ToF	0.64	0.50	0.97	0.82
	
	BAV	0.67			
	
	ASD/PAPVR	0.97	0.97	1.00	0.95

Correlation of stroke volume calculated for each vessel in each diagnostic group. RVOT/MPA = right ventricular outflow tract/main pulmonary artery, RPA = right pulmonary artery, LPA = left pulmonary artery, ToF = Tetrology of Fallot, BAV = bicuspid aortic valve, ASD = atrial septal defect and partial anomalous pulmonary venous return, All = combination of all diagnostic groups.

**Table 2:** Mean Difference in Stroke Volume (mL).

	Vessel SV (mL)			
	**Aorta**	**RVOT/MPA**	**RPA**	**LPA**

Mean	-7	7	-1	-2

-2SD	-25	-39	-9	-21

+2SD	30	52	6	17

Range of Absolute Difference	0-68	0-70	0-8	0-23

Differences in measured stroke volume without phantom correction minus measured stroke volume with phantom correction for all three diagnostic groups. RVOT/MPA = right ventricular outflow tract/main pulmonary artery, RPA = right pulmonary artery, LPA = left pulmonary artery.

### Shunt Calculation

We calculated Qp:Qs ratios using both SV and CO. A Qp:Qs ratio outside the range of 0.85-1.19 was considered to represent the presence of a shunt[13]. As shown in table 3, performing flow mapping with phantom correction caused 3 patients with ToF and 2 patients with ASD/PAPVR to change clinical classification. When calculating Qp:Qs ratios based on CO from flow mapping with phantom correction, no patients in the repaired ToF group and all of the patients in the ASD/PAPVR group had a significant shunt. This was not the case for flow mapping without phantom correction. When we averaged Qp:Qs ratios across diagnostic groups, the differences were not significant between the two methods. For the ToF group, the average Qp:Qs ratio was 1.18 (± 0.60) without and 1.02 (± 0.07) with phantom correction. In the ASD/PAPVR group, the Qp:Qs average was 1.74 (± 0.80) without and 1.76 (± 0.81) with phantom correction.

**Table 3:** Differences in Qp:Qs Shunt Classification.

		Calculations with SV			Calculations with CO		
		**Shunt**	**No Shunt**	**Change**	**Shunt**	**No Shunt**	**Change**

ToF	Phantom On	1 (8%)	12 (92%)	3 (23%)	0 (0%)	13 (100%)	3 (23%)
				
	Phantom Off	4 (31%)	9 (69%)		3 (23%)	10 (77%)	

ASD/PAPVR	Phantom On	10 (100%)	0 (0%)	2 (20%)	10 (100%)	0 (0%)	2 (20%)
				
	Phantom Off	8 (80%)	2 (20%)		8 (80%)	2 (20%)	

The number of patients (and percentage) with and without a shunt (as defined as less than 0.85 or greater than 1.19) is shown for patients with Tetrology of Fallot (ToF) and atrial level shunting (ASD/PAPVR). Qp is based on sum of branch pulmonary artery flow. SV = stroke volume. CO = cardiac output.

### Regurgitant Fraction

**Figure 2:**
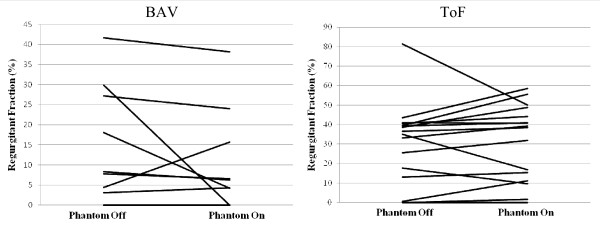
**Regurgitant Fraction**. Linear plots show the change in the calculated regurgitant fraction (RF) for each individual patient when flow mapping is performed with or without phantom correction. The graph on the left is representative of the RF in the aorta for patients with bicuspid aortic valve (BAV). The graph on the right represents the RF in the right ventricular outflow tract (RVOT) for patients with Tetrology of Fallot (ToF).

**Figure 3:**
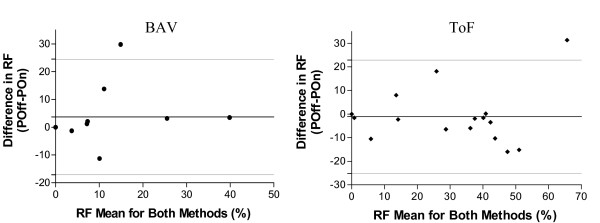
**Bland-Altman Analysis of Regurgitant Fraction Measurements with and without Phantom Correction**. Scatter plots show the mean regurgitant fraction (RF) compared to the difference in RF for measurements made with and without phantom correction. Each plot represents a single patient. The plot on the left represents the RF calculations for the aortic blood flow for patients with bicuspid aortic valve (BAV). The plot on the right represents RF calculations for flow through the right ventricular outflow tract for patients with Tetrology of Fallot (ToF). The solid horizontal line represents the mean of the differences in RF, while the dotted lines represent 2 standard deviations of that mean in each direction. Poff-Pon = RF measured without phantom correction minus RF measured with phantom correction.

We grouped our patients with BAV and ToF into standard regurgitant fraction (RF) classes of none (0%), mild (<20%), moderate (20-40%) and severe (>40%) to delineate the severity of RF across the aortic valve and RVOT, respectively. Analysis was performed based on flow mapping with and without phantom correction. As shown in table 4, 18% of the patients with BAV and 31% of patients with ToF changed their clinical classification depending on whether the flow mapping was performed with or without phantom correction. The mean absolute difference in RF when comparing the two methods was 6 ± 9% and 8 ± 9% for BAV and ToF, respectively. While this mean difference for the study populations was small, the difference for an individual could be large. Moreover the corrections, as shown in figure 2, were marked and variable at the individual level. Comparison of RF calculations by Bland-Altman analysis [12] in figure 3 shows that the variability between the two methods was high with 95% limits of agreement approaching a RF difference of 30%. The Bland-Altman analysis also suggests that there was no trend to correlate the magnitude of the RF and the variability in flow measurements with and without phantom correction.

**Table 4:** Differences in Regurgitant Fraction Classification.

		Regurgitant Fraction Classification				
		
		None	Mild	Moderate	Severe	Change
BAV, Aorta	Phantom On	4 (36%)	5 (45%)	2 (18%)	0 (0%)	2 (18%)
		
	Phantom Off	3 (27%)	5 (45%)	2 (18%)	1 (9%)	

ToF, RVOT	Phantom On	1 (6%)	5 (31%)	3 (19%)	7 (44%)	5(31%)
		
	Phantom Off	2 (13%)	3 (19%)	7 (44%)	4 (25%)	

The number of patients (and percentage) that have no (0%), mild (<20%), moderate (20-40%) or severe (>40%) regurgitant fraction (RF), based on flow mapping with (phantom on) or without (phantom off) phantom correction are shown. Change represents the number (and percentage) of patients that changed clinical classification when using the different methods of measurement. BAV = bicuspid aortic valve, ToF = Tetrology of Fallot, RVOT = right ventricular outflow tract.

## Discussion

While flow mapping by CMR has many advantages, it also has inherent sources of error. As stated above, many approaches are employed to compensate for baseline velocity errors. Options for correction include an estimate of background *flow *using a region of stationary tissue or estimating the spatial distribution of velocity variation using either linear or higher order interpolation functions. The limitations of such approaches have been previously reported [9-11]. Given these limitations we chose to apply phantom correction to flow mapping of patients with congenital heart disease. While the use of the phantom correction does add to scan time, it accurately measures background *flow *in the same location as the vessel in question and corrects for all elements that contribute to baseline shifts.

As the use of flow mapping in patients with congenital heart disease increases [14], the need for careful data acquisition and correction for background *flow *cannot be ignored. Failure to correct for such errors may lead to misinterpretation of data in individual patients. Our data clearly demonstrates that for any given patient with congenital heart disease, interpretation of cardiac physiology and pathology can be markedly different depending on whether flow mapping is done with or without phantom correction. This study did not try to address the causes of those differences, but rather highlight that they can be clinically significant in the congenital heart disease population and should not be ignored during data acquisition and analysis. At the same time, our data appears to support previously published reports which show where these eddy currents have a greater impact. Specifically, we found a greater discrepancy between flow measurements with and without phantom correction in the aorta and RVOT than in the BPAs. This is in agreement with the finding of Chernobelsky and colleagues in healthy volunteers that the size of the vessel and the magnitude of flow correction are related [7]. The orientation of the angle of the vessel in the magnet may also contribute to this difference but again the goal of our study was to emphasize the need for correction rather than the factors influencing the underlying error. Given the larger variation in individual patient anatomy and physiology in patients with repaired congenital heart disease (such as marked differences in the relative sizes of their aorta, main pulmonary artery and individual branch pulmonary arteries) even greater care is needed at the time of flow mapping assessment to minimize errors. The need to avoid other technical errors (including alignment with flow, partial volume averaging, appropriate Venc selection and avoiding non-laminar flow patterns) cannot be forgotten. We sought to minimize such additional sources of error by using 'standardized' protocols tailored to the patients' anatomy. As mentioned in the results section, 17% of flows were not accepted as accurate due to such technical issues.

One limitation of our study is the lack of a third, validated method (that measures true flow) with which to compare flow mapping with and without phantom correction. Without being able to define true flow, all of the data should be interpreted as if either mapping with or mapping without phantom correction could be the more accurate measurement. That said, we feel the former is more precise given that our calculated Qp:Qs ratios agreed more closely with what we would have expected clinically knowing the patients' echocardiographic data. In other words, with phantom correction repaired ToF patients had a Qp:Qs ratio closer to one, and all patients with unrepaired atrial level shunting had evidence of clinical shunting. While there are different standards on what ratio of pulmonary to systemic blood flow constitutes a clinically significant shunt, we classified our patients based on previously published CMR validation standards which found the Qp:Qs ratio in 20 pediatric patients without a shunt to range from 0.85-1.19 [13]. Admittedly, if we were to use a broader definition of clinically significant shunt (such as Qp:Qs >1.5), fewer patients would have changed clinical classification. The use of a narrower range, however, determines if there is a small shunt even if no therapeutic intervention is indicated. A second limitation of our study is that the two magnets (identical models) used in this study were from a single vendor. There is no doubt that variability from magnet to magnet can occur but also that variation in errors can differ by vendor, model and site [15]. That said, one clear benefit of stationary phantom correction is that its application is independent of magnet type and vendor. Finally, our study is limited by size. A larger study population may have provided enough power to make the difference in Qp:Qs ratios between the two methods significant. Larger numbers would also have allowed for subgroup analysis between the two magnets used at our institution. This may be a source of future research at a multi-center level [15].

## Conclusions

The accuracy of flow mapping by CMR is significantly affected by baseline velocity offsets from multiple sources including eddy currents. In patients with congenital heart disease, significant errors in flow quantification may occur when such offsets are not accounted for. The use of a stationary phantom allows for correction of background *flow *errors due to phase shifts. The correlation of flow measurements with and without phantom correction appears to be highly variable and unpredictable in patients with congenital heart disease. As a result, the use of phantom correction appears to be a useful and easily implemented correction that can lead to marked changes in the clinical interpretation of flow data in such patients.

## Competing interests

The authors have no financial or non-financial competing interests to disclose. The above results were presented, in part, at the 12^th ^annual scientific sessions of the Society for Cardiovascular Magnetic Resonance.

## Authors' contributions

TAM performed all of the retrospective analysis of flow mapping with and without phantom correction, performed all data and statistical analysis and drafted the manuscript. ABL and AMM conceived and designed the study, participated in review of data analysis and helped draft the manuscript. ABL and AMM are also involved in direct oversight of all CMR performed at our institution.

## Authors' Information

TAM is a pediatric resident. ABL and AMM are the co-directors of Maine Medical Center's cardiac magnetic resonance program. They are attending physicians in pediatric radiology and pediatric cardiology, respectively. AMM is also the director of pediatric cardiology at Maine Medical Center.
